# Role of Sterylglucosidase 1 (Sgl1) on the pathogenicity of *Cryptococcus neoformans*: potential applications for vaccine development

**DOI:** 10.3389/fmicb.2015.00836

**Published:** 2015-08-11

**Authors:** Antonella Rella, Visesato Mor, Amir M. Farnoud, Ashutosh Singh, Achraf A. Shamseddine, Elitza Ivanova, Nicholas Carpino, Maria T. Montagna, Chiara Luberto, Maurizio Del Poeta

**Affiliations:** ^1^Department of Molecular Genetics and Microbiology, Stony Brook University, Stony BrookNY, USA; ^2^Department of Medicine, Stony Brook University, Stony BrookNY, USA; ^3^Department of Biomedical Science and Human Oncology, Hygiene Section, University of BariBari, Italy; ^4^Department of Physiology and Biophysics, Stony Brook University, Stony BrookNY, USA

**Keywords:** yeast genetics, gene expression, glycolipid, immunosuppression, vaccine development

## Abstract

Cryptococcosis caused by *Cryptococcus neoformans* and *Cryptococcus gattii* affects a large population and is a cause of significant morbidity and mortality. Despite its public health burden, there are currently no vaccines against cryptococcosis and new strategies against such infections are needed. In this study, we demonstrate that *C. neoformans* has the biochemical ability to metabolize sterylglucosides (SGs), a class of immunomodulatory glycolipids. Genetic manipulations that eliminate cryptococccal sterylglucosidase lead to the accumulation of SGs and generate a mutant strain (Δ*sgl1*) that is non-pathogenic in the mouse models of cryptococcosis. Interestingly, this mutant strain acts as a vaccine strain and protects mice against cryptococcosis following infection with *C. neoformans* or *C. gattii*. The immunity induced by the Δ*sgl1* strain is not CD4^+^ T-cells dependent. Immunocompromised mice, which lack CD4^+^ T-cells, are able to control the infection by Δ*sgl1* and acquire immunity against the challenge by wild-type *C. neoformans* following vaccination with the Δ*sgl1* strain. These findings are particularly important in the context of HIV/AIDS immune deficiency and suggest that the Δ*sgl1* strain might provide a potential vaccination strategy against cryptococcosis.

## Introduction

*Cryptococcus* is an opportunistic fungal pathogen and the causative agent of the disease cryptococcosis. Infections caused by *Cryptococcus neoformans* and *Cryptococcus gattii* lead to more than 600000 deaths per year (Park et al., 2009), especially among immunocompromised individuals. Despite its significant public health burden, no vaccines currently exist in the clinic for cryptococcosis (or other fungal infections Nanjappa and Klein, 2014). Although experimental vaccines have been developed using the glucuronoxylomannan (GXM) capsule bound to tetanus toxoid (Devi et al., 1991; Casadevall et al., 1992; Devi, 1996), these formulations have not been translated to the clinic and have suffered from drawbacks such as inducing detrimental antibodies in mice (Casadevall and Pirofski, 2005; Datta and Pirofski, 2006). Recent attempts in the mouse models of cryptococcosis have been focused on the use of genetically engineered *C. neoformans* strains that generate cytokines (Wormley et al., 2007; Wozniak et al., 2011) or protein preparations from *C. gattii* administered prior to infection (Chaturvedi et al., 2014). Although these attempts have provided valuable insights, studies are still limited and shortcomings exist. For example, complete immunity against *C. gattii* (responsible for severe infections in the USA Datta et al., 2009; Walraven et al., 2011) has not been achieved (Chaturvedi et al., 2014) demonstrating the need for the development of more effective vaccines.

Sterylglucosides (SGs) are a class of glycolipids produced by animals, plants, bacteria and various fungi including *C. neoformans* (Warnecke et al., 1999; Watanabe et al., 2015). SGs show immunomodulatory properties as reported by various studies. For example, SGs have been shown to increase proliferation of lymphocytes and eosinophils *in vivo* (Donald et al., 1997) and cytokine secretion *in vitro* (Bouic et al., 1996; Lee and Han, 2006; Lee et al., 2007). In the realm of fungal infections, it has been observed that SGs administration increases the survival of mice infected with a lethal dose of *Candida albicans* by inducing a Th1 immune response (Lee et al., 2007).

A recent report has identified a endoglycoceramidase-related protein (named EGCrP2), capable of hydrolyzing SGs in *C. neoformans* (Watanabe et al., 2015). We hypothesized that the lack of this enzyme should lead to a lack of SG catabolism and subsequent SG accumulation, allowing for the immunomodulatory properties of SGs to manifest. Thus, we sought to utilize the immunomodulatory properties of fungal SGs by engineering a *C. neoformans* strain that lacks the sterylglucosidase enzyme. This strain was non-pathogenic in the mouse models of cryptococcosis and conferred complete protection against both *C. neoformans* and *C. gattii*, suggesting that it might be a suitable candidate for vaccine development against cryptococcosis.

## Materials and Methods

### Strains, Plasmid, and Culture Conditions

The fungal strains used in this study were *C. neoformans* (*Cn*) var. *grubii* strain H99 wild-type (WT) and *C. gattii* strain R265 and *Saccharomyces cerevisiae* ΔYIR007W mutant (*Sc*ΔYIR) derived from BY4741. Bacterial strain *Escherichia coli* DH5-α (Life Technologies, Carlsbad, CA, USA) was used as competent cells. The plasmid pCR II-TOPO 4.0 kb was used for cloning, and pYES2/CT was used for expression of *Cn* 5607 in *Sc*ΔYIR.

Bacterial strains were grown at 37°C in Luria-Bertani medium with 75 mg/L of ampicillin. *Cn* strains were routinely grown in YPD broth at 30°C and 0.04% atmospheric CO_2_ for 20–22 h with shaking at 250 rpm. Dulbecco’s modified eagle media (DMEM) buffered with 25 mM HEPES and 2% glucose at pH 7.4 or pH 4 was used for growing *Cn* at 37°C and 5% CO_2_ (i.e., physiologically relevant conditions). *Sc*ΔYIR strain transformed with pYES2/CT or pYES2/CT- *Cn* 5607, was grown in yeast nitrogen base (YNB) without amino acids, 1.2 g/L amino acid mixture lacking uracil (ura-), and 2% glucose or 2% galactose (48 h) at 30°C to induce *Cn* 5607 expression.

### Expression of *Cn* 5607 in *S. cerevisiae*

*Cn* 5607 was identified by blasting the *Cn* WT endoglycoceramidase-related Protein 1 (*EGCrP1*) sequence in *Cn* WT Broad Institute genome database [http://www.broadinstitute.org/annotation/genome/cryptococcus_neoformans/MultiHome.html]. Two sequences were found: one 100% identical to *EGCrP1* and another one with an E-value of 9e-57, located on chromosome 14 (*Cn* 5607). To express *Cn* 5607 in *S. cerevisiae* strains, total RNA was extracted from *Cn* and the cDNA was synthesized from 1 μg of the total RNA using SuperScript III RNase H-Reverse Transcriptase (Life Technologies). PCR was performed using the cDNA as a template and the following expression primers: PR*SGL1*-5′ forward (5′-GAGCTCATGCCTCCTCCACCAGAAGT-3′) and PR*SGL1*-5′ reverse (5′-TCTAGAAGCAATAACGCATTCAGGACA-3′) carrying the restriction enzyme sites for SacI and XbaI respectively. A 2,556-pb fragment was cloned into pCR II-TOPO vector generating plasmid pCR- *Cn* 5607 and sequenced. After digestion with SacI and XbaI, *Cn* 5607 was inserted into pYES2/CT vector generating pYES2/CT-*Cn* 5607. *Sc*ΔYIR was grown in YNB medium overnight at 30°C and transformed with pYES2/CT empty-vector or pYES2/CT-*Cn* 5607 using lithium acetate transformation, as previously described (Kawai et al., 2010). After transformation, cells were plated on YNB ura- plates and incubated for 2–3 days in 30°C incubator. Then, *S. cerevisiae* colonies were patched with sterile toothpicks to fresh YNB ura- plates. To verify the expression of *Cn* 5607, one single colony containing pYES2/CT or pYES2/CT-*Cn* 5607 construct was inoculated into 10 ml of YNB ura- containing 2% of glucose and was grown overnight at 30°C with shaking. The cells were washed twice with PBS 1X and a suitable amount of overnight culture necessary to obtain an OD_600_ of 0.4 was transferred to a fresh YNB ura- medium containing 2% of galactose (induction medium) and incubated for 48 h at 30°C with shaking. After 48 h, the cells were washed and harvested by centrifugation at 3000*g* for 5 min at 4°C, and the cell pellets was stored at -80°C until ready to be used.

Total proteins were extracted from *S. cerevisiae* strains as previously described (Singh et al., 2011). Protein content was assessed by the method of Bradford using bovine serum albumin (Sigma-Aldrich, St. Louis, MO, USA) as a standard. Fifty micrograms of *S. cerevisiae* proteins were loaded onto SDS-PAGE and stained with Coomassie Brilliant Blue. Western Blot was used to detect the expression of recombinant fusion protein using Anti-His (C-term)-HRP antibody.

### *In Vitro* Activity Assay of *Cn* 5607 Using Standard Plants SGs and Purified *Cn* SGs

The *in vitro Cn* 5607 enzymatic assay was performed using 20 μg of standard SGs (purified from plants and commercially available) or 10 μg of endogenously purified SGs as substrate and *Sc*ΔYIR + empty vector or *Sc*ΔYIR*-Cn* 5607 as source of enzyme. The decrease in the intensity of the SGs band was monitored by thin layer chromatography (TLC). Plants SGs were incubated with 200 μg of *Sc*ΔYIR + empty vector or 50, 100, and 200 μg of ScΔYIR-*Cn* 5607 at 30°C for 1 h. Endogenously purified *Cn* SGs were incubated with 200 μg of *Sc*ΔYIR + empty vector or 100 μg of ScΔYIR-*Cn* 5607 at 30°C for 1 h. The reactions were terminated by the addition of 300 μl of CHCl_3_/MeOH (1:1 ratio), the lower phases were dried down, resuspended in 50 μl of CHCl_3_/MeOH (2:1 ratio) and analyzed by TLC. *Cn* 5607 specifically cleaves SGs, thus we called *Cn* 5607 sterylglucosidase1 (Sgl1).

The pH dependence of *Sgl1* was determined using as substrate SGs in a pH range of 4.5–8 using the following buffers at the final concentration of 50 mM: sodium acetate (pH 4.5–5.0), MES (pH 5.5–6.0), sodium phosphate (pH 6.5–7.0), and HEPES (pH 7.5–8.0). The optimal temperature of *Sgl1* was determined in the range from 25 to 37°C. The effect of detergents was assessed using Triton X-100, Sodium Deoxycholate and CHAPS at the concentration of 0.05, 0.15, and 0.3%.

### *In Vitro* Activity Assay of *Cn* 5607 Using NBD-C_6_-Glucosylceramide and *Cn* Long Chain GlcCer

To verify the enzymatic activity of *Cn* 5607, we used different substrates: NBD-C_6_-glucosylceramide (NBD-C_6_-GlcCer; Matreya, LLC, State-College, PA, USA) and *Cn* long chain GlcCer. Briefly, 200 μg of yeast proteins from *Sc*ΔYIR + empty vector or *Sc*ΔYIR-*Cn* 5607 were incubated first with 20 μM of NBD-C_6_-Glucosylceramide and at 30°C for 1 h in a final reaction volume of 100 μl. The production of NBD-C_6_-Ceramide was identified as a fluorescent band using a PhosphorImager^TM^ 860 STORM unit and ImageQuant analysis (GE Healthcare, Rahway, NJ, USA) as previously described (Rittershaus et al., 2006).

The *in vitro Cn* 5607 activity was also valuated using 10 μg of *Cn* long chain GlcCer and 200 μg of *Sc*ΔYIR + empty vector or *Sc*ΔYIR-*Cn* 5607 cell extracts. Cerezyme (10 μg), generously provided by the Genzyme Corporation (Cambridge, MA, USA), was used as positive control for the catalytic reaction. The reactions were terminated by the addition of 300 μl of CHCl_3_/MeOH (1:1 ratio), the samples were mixed and the phases were separated by centrifugation at 3000*g* for 5 min. The lower phases were dried down using a SPD 2010 SpeedVac vacuum dryer (Thermo Electron Corp.). The dried samples were resuspended in 50 μl of CHCl_3_/MeOH (2:1 ratio) and analyzed by TLC on silica gel plate (EMD Millipore, Billerica, MA, USA) developed with chloroform/methanol/water (65:25:4, v/v/v) and stained with iodine and resorcinol. The *in vitro* activity assay using *Cn* long chain GlcCer and *Sc*ΔYIR + empty vector or *Sc*ΔYIR-*Cn* 5607 was also repeated with a longer incubation time (4 h) and the results were evaluated by liquid chromatography–mass spectrometry (LC–MS).

### Disruption and Reconstitution of *SGL1* Gene in *Cn*

The *SGL1* gene (locus number CNAG_05607 in *C. neoformans* var. *grubii* serotype A genome database) was deleted using NAT1 (Nourseothricin Acetyl transferase1) split marker. A knockout cassette was generated containing a 1.035 bp of the 5′ untranslated region (5′UTR) upstream of the ATG start codon of the S*GL1* gene and a 1.059 bp of the 3′UTR. The 5′UTR was amplified by PCR using H99 genomic DNA as a template and the following primers: 5′UTR-F (5′-GTCAAGCTAAGAGCTCCATTTGATCAGCGGGATTCT-3′) and 5′UTR-R (5′-TCCACTCCGAACTAGTATCGCGTAAACGAAGAGGTG-3′), containing SacI and SpeI sites, respectively (underlined). The 3′UTR was amplified by PCR using H99 genomic DNA as a template and the following primers: 3′UTR-F (5′-GTCAAGCTAATCTAGAAGCCCATTCTGGTTGTTCTG-3′) and 3′UTR-R (5′-ACATCACACTTCTAGATTTAGCGAGCCACGTTTTCT-3′). The amplified fragments were cloned in pCR II-TOPO and sequenced, generating plasmid pCR-5′UTR-TOPO and pCR-3′UTR-TOPO. *NAT1* gene, which confers resistance to the antibiotic nourseothricin (Werner BioAgents, Jena, Germany), under the control of *Cn* actin promoter was digested from the plasmid pCR-NAT1-TOPO by SacI and SpeI and ligated with 5′UTR digested with the same restriction enzyme generating pCR-5′UTR-NAT1-TOPO. Finally, pCR-5′UTR-NAT-TOPO was digested by EcoRV and ligated with 3′UTR generating the disruption cassette pCR-5′UTR-NAT1-3′UTR-TOPO that was named pΔ*sgl1*. The deletion scheme is illustrated in Supplementary Figure S3A. *Cn* WT was transformed with the plasmid pΔ*sgl1* using biolistic DNA delivery device, as described previously (Toffaletti et al., 1993). Stable transformants were grown on YPD plates containing 100 μg/ml of nourseothricin. Colonies were chosen randomly and genomic DNA was isolated and digested with EcoRV and KpnI for Southern blot analysis. The DNA fragments were screened by probing with a fragment of 5′UTR. Transformant #106 showing deletion of the *SGL1* gene by insertion of the *NAT1* was chosen and designated Δ*sgl1* mutant strain. *SGL1* gene was reintroduced back into the Δ*sgl1* using the reconstitution cassette pSK-*SGL1*-HYG, which had the Hygromycin B allele as selectable marker. The reconstitution scheme is illustrated in Supplementary Figure S3B. The plasmid pSK-*SGL1*-HYG was biolistically delivered into Δ*sgl1.* Homologous recombinants were screened by Southern hybridization using a 800 bp fragment of the *SGL1* open reading frame as probes. Transformant #21 showing reconstitution of *SGL1* gene was designated *Δsgl1+SGL1* reconstituted strain.

Wild-type, mutant, and reconstituted strains were characterized for their growth profile, capsule formation, stress response, and intracellular growth. For growth profile studies, WT, Δ*sgl1*, and Δ*sgl1+SGL1* reconstituted strains were grown overnight in YPD at 30°C, the cells were washed three times with PBS, counted, and diluted to a final density of 10^4^ cells/ml in DMEM at pH 7.4 or pH 4 and incubated at 37°C in the presence of 5% CO_2_. Aliquots were taken at different time points, diluted, and plated in duplicates onto YPD agar plates for assessment of CFUs. Capsule thickness and melanin production were determined as previously described (Wang et al., 1995; Shea et al., 2006). For oxidative stress studies, strains were spotted in serial dilution (10^7^, 10^6^, 10^5^, 10^4^, 10^3^) on YPD agar plates with 25 mM HEPES (pH 7 or pH 4) supplemented with 5 mM H_2_O_2_, cells growth was assessed after incubation at 30°C for 96 h. Nitrosative stress response was studied by spotting the strains in serial dilution (10^7^, 10^6^, 10^5^, 10^4^, 10^3^) on YNB agar plates with 25 mM succinate acid (pH 4) supplemented with 0.1 mM NaNO_2_. Cell growth was assessed after 96 h of incubation at 30, 37°C in atmospheric environment or 37°C in the presence of 5% CO_2_.

Phagocytosis and intracellular killing studies were performed in J774.16 macrophage-like cells as previously described (Tripathi et al., 2012). Briefly, for phagocytosis experiments cells were plated in a 96 well plate in Dulbecco’s minimal essential medium (DMEM) supplemented with 10% fetal bovine serum (FBS). *C. neoformans* cells were grown overnight in YPD at 30°C. Cells were washed twice in PBS and counted. Approximately 10^5^ cells in DMEM + FBS medium were opsonized with 10 μg/ml of anti-GXM monoclonal antibody 18B7 (kindly provided by Dr. Arturo Casadevall) and added to macrophage-like cells activated with 50 units/ml of recombinant murine gamma interferon and 0.3 μg/ml of lipopolysaccharide at an effector-to-target ratio of 1:1. After incubation for 2 h, extracellular *C. neoformans* cells were washed with three changes of warm DMEM medium and fresh medium. Then, 200 μl of sterile water was added to each well and the macrophage-like cells were lysed by pipetting several times. CFUs were analyzed by plating them on YPD agar plates and the numbers of internalized fungal cells were reported. Intracellular killing were performed in the same way with the following change: extracellular *C. neoformans* cells were washed off once 2 h after the initial incubation and another time after 24 h of incubation. Macrophage-like cells were lysed after 24 h by pipetting several times and CFUs were analyzed by plating them on YPD agar plates.

### Lipid Analysis of *Cryptococcus* Strains by TLC

Total lipids from *Cryptococcus* strains were extracted, as described previously (Singh et al., 2012). Briefly, a single colony of *Cryptococcus* strains was grown in 15 ml of YPD broth at 30°C for 20 h at 250 rpm. *Cryptococcus* cells (5 × 10^8^) were placed in a single glass tube to which the Mandala extraction buffer was added (Mandala et al., 1995). Lipid extraction was performed according to the methods of Bligh and Dyer (Bligh and Dyer, 1959) followed by base hydrolysis. One set of dried samples was resuspended in 50 μl of CHCl_3_/MeOH (2:1 ratio) and analyzed by TLC developed with chloroform/methanol/water (65:25:4, v/v/v) and stained with iodine and resorcinol, the other set was used for gas chromatography–mass spectrometry (GC–MS).

### Lipid Profiling by Mass Spectrometry

Total lipids were extracted from *Cryptococcus* strains, using the methods described previously (Singh et al., 2012). For sterylglucosides analyses, extracted lipid samples were derivatized using *N, O*-bis (trimethylsilyl) trifluoroacetamide/trimethylchlorosilane reagent (Sigma-Aldrich) and then analyzed using 30 mt (0.25 μm) DB5-MS column on Agilent 7890 GC–MS (Agilent Technologies, Santa Clara, CA, USA). The retention time and mass spectral patterns of plant SGs standard (Avanti Polar Lipids, Inc., Alabaster, AL, USA) were used as a reference (Gutierrez and del Rio, 2001). Cholesterol was added as an internal standard for these analyses prior to lipid extraction. Ceramide and glucosylceramide species were analyzed by multiple reactions monitoring (MRM) as described previously (Singh et al., 2012) using TSQ Quantum Ultra^TM^ Triple Quadrupole Mass Spectrometer (Thermo Scientific, USA). Samples were delivered by Accela pump (Thermo Finnigan, USA) to the HPLC fitted with 3 μm C8SR, 150 mm × 3.0 mm column (Peeke Scientific, Sommerset, NJ, USA). C17 sphingosine and C17 ceramide were added as an internal standard for these analyses prior to lipid extraction. Determination of plant sterols and sterylglucosides for enzymatic activity assay was performed using MRM monitoring on LC–MS (Wewer et al., 2011). Standard plant sterols and sterylglucosides (Avanti Polar Lipids, Inc.) were used as the external standards in these measurements.

### Animal Studies

Four weeks old female CBA/J mice (Harlan Laboratories, Indianapolis, IN, USA) were used for all studies. Mice were anesthetized with an intraperitoneal injection of 60 μl xylazine/ketamine mixture containing 95 mg ketamine and 5 mg xylazine per kilogram of body weight and infected. For the infection studies, 24 mice (eight for each group) were infected intranasally with 5 × 10^5^ cells/20 μl of WT, Δ*sgl1* or Δ*sgl1+SGL1* reconstituted strain. Mice were inspected twice a day and those that appeared moribund or in pain were sacrificed with CO_2_ inhalation followed by cervical dislocation. All animal procedures were approved by Stony Brook University Institutional Animal Care and Use Committee and followed the guidelines of American Veterinary Medical Association. For tissue burden analysis, four mice per strain were used. Lung, brain, liver, kidney and spleen were excised and homogenized in 10 ml of PBS using Stomacher 80 (Seward, UK) for 2 min at high speed. Several dilutions were plated in duplicate onto YPD agar plates and incubated for 48–72 h at 30°C. The CFUs per organ were counted. For histopathology analysis, three mice per strain were used. Mice organs were fixed in 3.7% of formaldehyde in paraffin and stained with haematoxylin and eosin and mucicarmine.

For *in vivo* vaccination studies, mice were pre-treated with vehicle (PBS), Δ*sgl1* (5 × 10^5^ cells), and Δ*gcs1* (5 × 10^5^ cells). After 30 days, mice pre-treated with vehicle or Δ*sgl1* were challenged with 5 × 10^5^ cells of *Cn* WT or *Cg* R265. Mice pre-treated with Δ*gcs1* were challenged with 5 × 10^5^ cells of *Cn* WT. Mouse survival was monitored for 80 days after post-challenge. CD4^+^ T-cell depletion was achieved by weekly intraperitoneal administration of anti-CD4^+^ (GK1.5, rat IgG2b, 200 μg in 200 μL of PBS; National Cell Culture Center, Minneapolis, MN, USA). A rat IgG2b (eBioscience, Inc., San Diego, CA, USA) was used as control. T-cell depletion was assessed by flow cytometry in the spleens. For vaccination studies, mice (eight for each group) were pre-treated with vehicle (PBS) or Δ*sgl1* strain after 48 h from the first round of T-cell depletion and after 30 days were challenged with a lethal dose of *Cn* WT (5 × 10^5^ cells) and their survival was monitored for 80 days.

## Results

### CNAG_05607 has Sterylglucosidase and not Glucosylceramidase Activity

**FIGURE 1:**
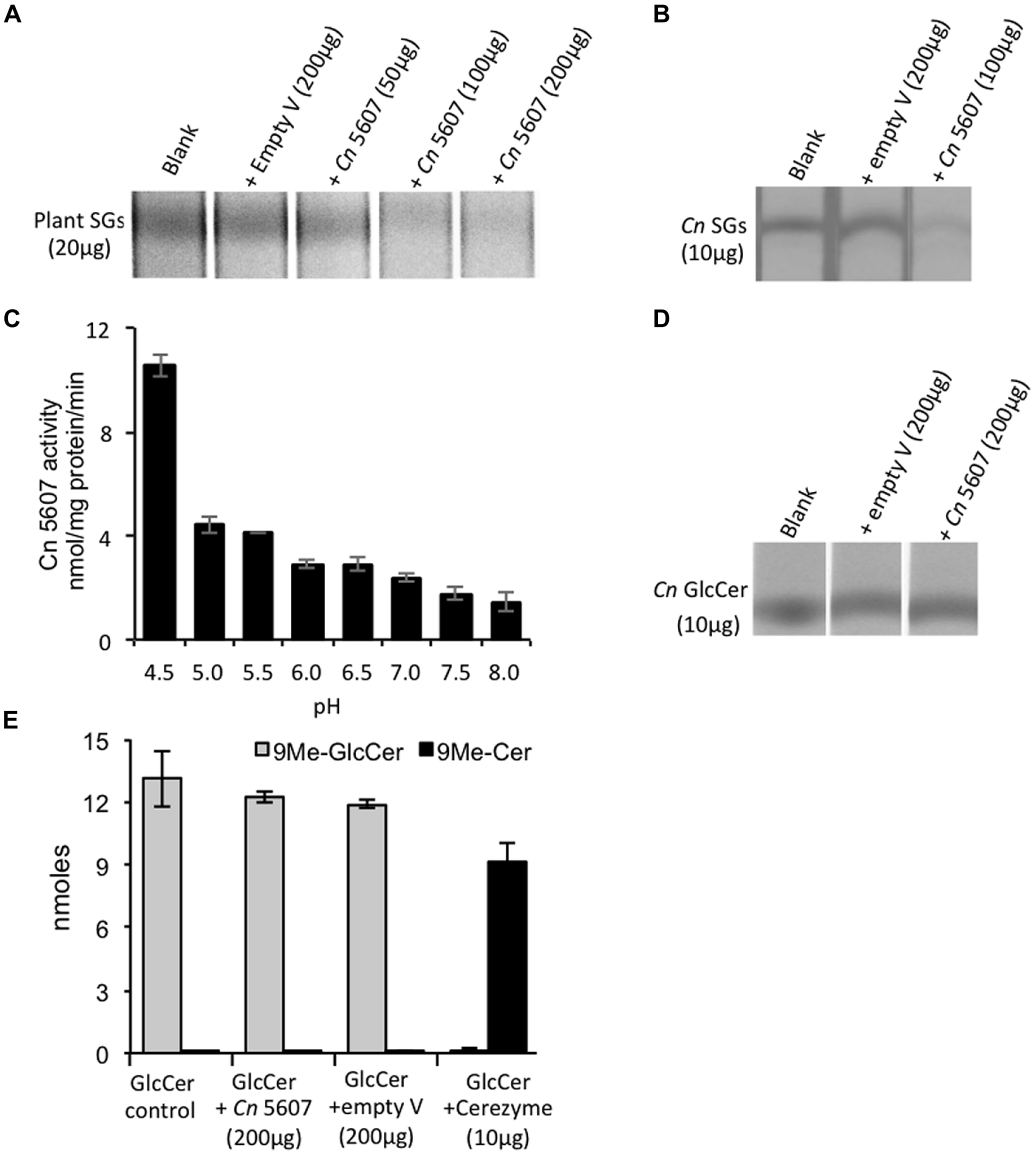
**CNAG_05607 enzyme has sterylglucosidase and not glucosylceramidase activity. (A)** *Cryptococcus neoformans* CNAG_05607 (*Cn* 5607) metabolizes plants SGs in a dose dependent manner and **(B)** metabolizes SGs extracted from *Cn* cells (*Cn* SGs). **(C)** pH dependence of *Cn* 5607 activity, as measured based on its ability to cleave plants SGs and produce free sterols, showed maximal activity at pH 4.5. Results are based on the measurement of free sterols performed by liquid chromatography–mass spectrometry (LC–MS). *Cn* 5607 does not metabolize *C. neoformans* GlcCer (*Cn* GlcCer) as analyzed by **(D)** thin layer chromatography (TLC) or by **(E)** LC–MS (Cerezyme is an analog of the human enzyme β-glucocerebrosidases, and was used as a positive control). *Cn* 5607 is the endoglycoceramidase-related protein 2 (EGCrP2) also identified by Watanabe et al. (2015). Empty V, empty vector.

A *S. cerevisiae* expression system was used for characterizing the activity of the CNAG_05607 enzyme. The blast search of CNAG_05607 in *Saccharomyces* genome database revealed a gene YIR007W with an identity of 41% (expect = 1.8e-129) to CNAG_05607 (Supplementary Figure S1). Therefore, a *Sc*ΔYIR mutant strain lacking of YIR007W gene was used for the studies. CNAG_05607 was cloned in pYES/CT vector and overexpressed in *S. cerevisiae* YIR007W mutant strain (ScΔYIR + *Cn* 5607). As a negative control, *Sc*ΔYIR mutant was transformed with pYES/CT empty vector (*Sc*ΔYIR + empty vector). Total proteins were extracted from *S. cerevisiae* strains, which contained the empty vector (control) or overexpressed the CNAG_05607 enzyme, and were incubated with plant (**Figure 1A**) or cryptococcal sterylglucosides (SGs; **Figure 1B**). With either substrate, 100 μg of total protein extract was enough to significantly degrade the SGs as evidenced by the disappearance of the SGs band on the TLC. No difference in the intensity of the SGs band was detected compared to the SG control when *Sc*ΔYIR mutant strain carrying the empty vector was incubated with plant or cryptococcal SGs. The activity of the enzyme was dependent on pH (**Figure 1C**) and temperature (data not shown), with the maximum activity observed at a pH 4.5 in sodium acetate buffer and a temperature of 37°C. In addition to cryptococcal SGs, the CNAG_05607 enzyme was also able to degrade cholesterol glucoside, the mammalian form of SGs (Supplementary Figure S2).

The CNAG_05607 enzyme has recently been characterized as a glucosylceramidase due to its ability to hydrolyze short-chain glucosylceramides (Watanabe et al., 2015). Our initial biochemical characterization also showed that CNAG_05607 metabolizes short chain glucosylceramide (data not shown) similarly to what was observed by Watanabe et al. (2015). However, CNAG_05607 did not metabolize long-chain, physiologically relevant, Δ8-C9 methyl glucosylceramides (**Figures 1D,E**), which is the form of glucosylceramide found in *C. neoformans.* To the best of our knowledge, glucosylceramide synthase and glucosylcerebrosidase, do not need a co-factor or activator to exert their activity on long chain GlcCer (i.e., C16 GlcCer; Akiyama et al., 2013). Cerezyme, a human recombinant glucosylcerebrosidase, was used as control. This enzyme metabolized NBD-C_6_-GlcCer (data not shown) as well as long-chain *Cryptococcus*Δ8-C9 methyl glucosylceramides resulting in ceramide production (**Figure 1E**). Cerezyme did not exhibit activity on plants or cryptococcal SGs (data not shown). Thus, these results demonstrate that CNAG_05607 has specific activity toward sterylglucosides, therefore we re-named this enzyme Sterylglucosidase 1 (Sgl1).

### Deletion of *SGL1* Causes Accumulation of SGs and not GlcCer

**FIGURE 2:**
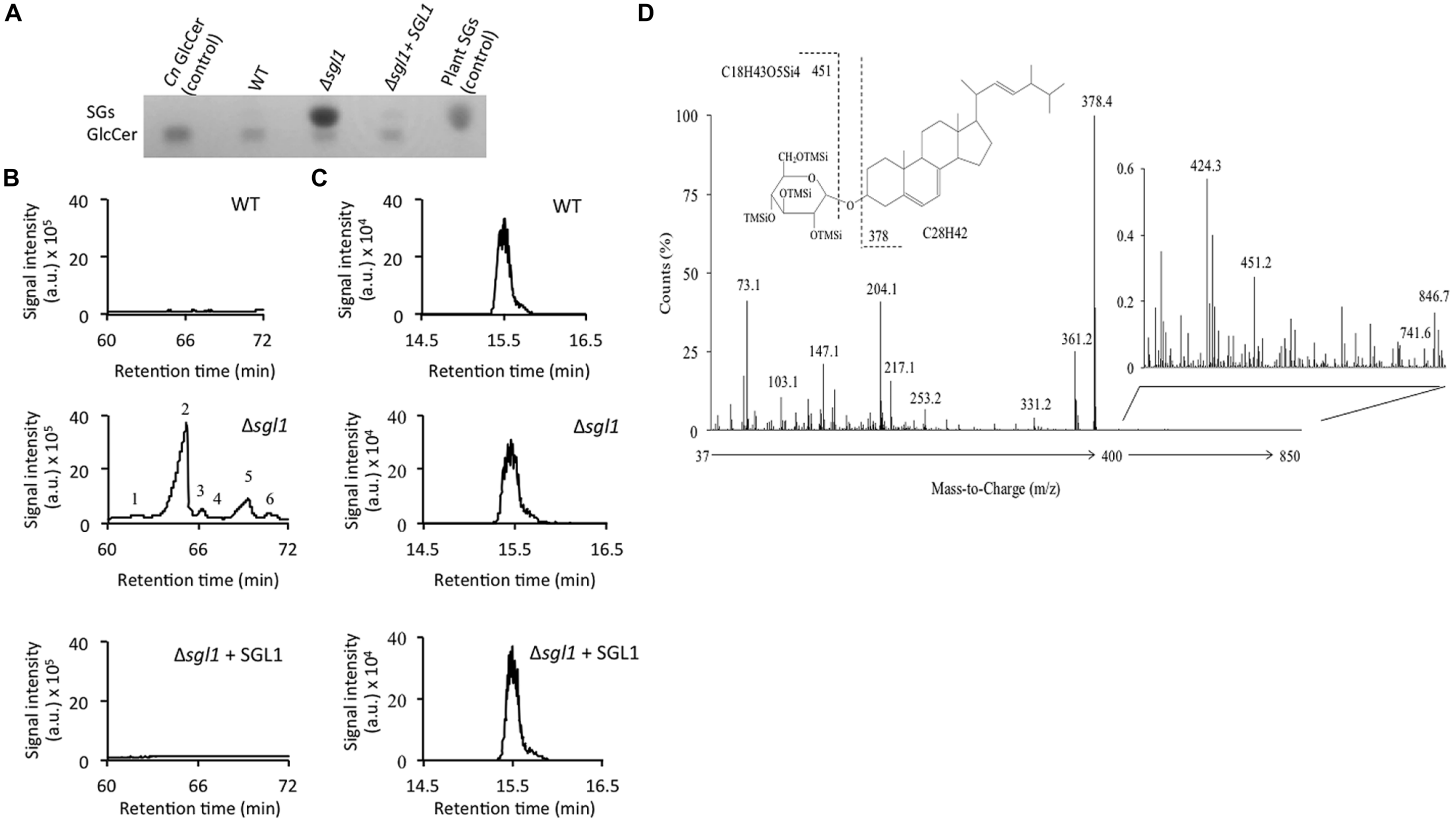
**Deletion of *SGL1* causes accumulation of SGs and not GlcCer.** Analysis of sterylglucosides (SGs) and glucosylceramide (GlcCer) was performed by **(A)** TLC and **(B,C)** gas chromatography-mass spectrometry. Results show that the Δ*sgl1* mutant dramatically accumulates SGs **(A,B)**, which are normally undetectable in wild-type (WT) or reconstituted strain (Δ*sgl1+ SGL1*). In contrast, the level of GlcCer **(A,C)** in the Δ*sgl1* mutant is identical to the one observed in the WT or Δ*sgl1+SGL1* reconstituted strain. Chromatograms are representatives of three separate experiments showing similar results. Peaks denote: (1) Dehydroergosteryl-β-D-glucoside^∗^; (2) Ergosteryl-β-D-glucoside; (3) Ergosta-7,22-dien-3-oyl-β-D-glucoside^∗^; (4) Fecosteryl-β-D-glucoside^∗^; (5) Episteryl-β-D-glucoside^∗^; (6) Lanosteryl-β-D-glucoside^∗^ (^∗^: putative structures). **(D)** Structure and electron-impact (EI) mass spectrum of peak number 2.

Since the Sgl1 enzyme acts to metabolize cryptococcal SGs, deletion of this enzyme in *C. neoformans* should lead to a SGs accumulating strain. This hypothesis was tested by genetically eliminating the sterylglucosidase enzyme (Supplementary Figure S3) in *C. neoformans* and monitoring the lipid profile by performing TLC and GC–MC on the total lipids extracted from the WT and the mutant strain. It was found that while the WT *C. neoformans* produces very little SGs, genetic elimination of sterylglucosidase (the Δ*sgl1* mutant) leads to a dramatic SGs accumulation; a phenomenon that is restored in the reconstituted strain (Δ*sgl1+SGL1*; **Figures 2A,B**). In agreement with the *in vitro* activity studies, elimination of sterylglucosidase did not affect glucosylceramide levels in the cell (**Figure 2C**), further confirming the sterylglucosides-specific activity of this enzyme.

In depth analysis of the MS spectrum of Δ*sgl1* strain showed the accumulation of 9 structures (**Figure 2B**) with ion fragments of m/z 147, 204, 217, 305, 361, 451. These structures were characteristic of tetramethylsilyl (TMSi) glucose ion fragments resulting from cleavage of C–O bonds. The fragments with m/z 361 and 451 are representative of TMSi derivative of hexoses. Ion fragments with m/z 129 and 255, characteristic of steroid moiety, were also present. Ion fragments with m/z of 73 and 147 represent the cleavage of 1 TMSi and 2 TMSsi groups respectively. The signal intensity of ion m/z 204 was greater than 217, which represented pyranoside configuration of the *O*-glycosidic linkage. These ion fragments resembled the fragmentation pattern generated during the MS analysis of plant sterylglucosides (Gutierrez and del Rio, 2001). Altogether, the ion fragments analysis confirmed that the structure possessed all characteristics of sterylglucosides. One of the most accumulated structures in the Δ*sgl1* mutant was ergosterolglucoside (Peak 2, **Figure 2B**). Apart from other characteristic ion fragments of sterolglucoside, MS fragmentation of peak 2 showed an ion fragment of m/z 378, which results from the cleavage C–O linkage of *O*-linked glucose moiety and is characteristic to ergosterol, suggesting that ergosterolglucoside was the structure with the highest concentration in the Δ*sgl1* strain. The chemical structure and the electron-impact mass spectrum of this molecule is presented in **Figure 2D**.

### Sgl1 is a Virulence Factor of *C. neoformans*

**FIGURE 3:**
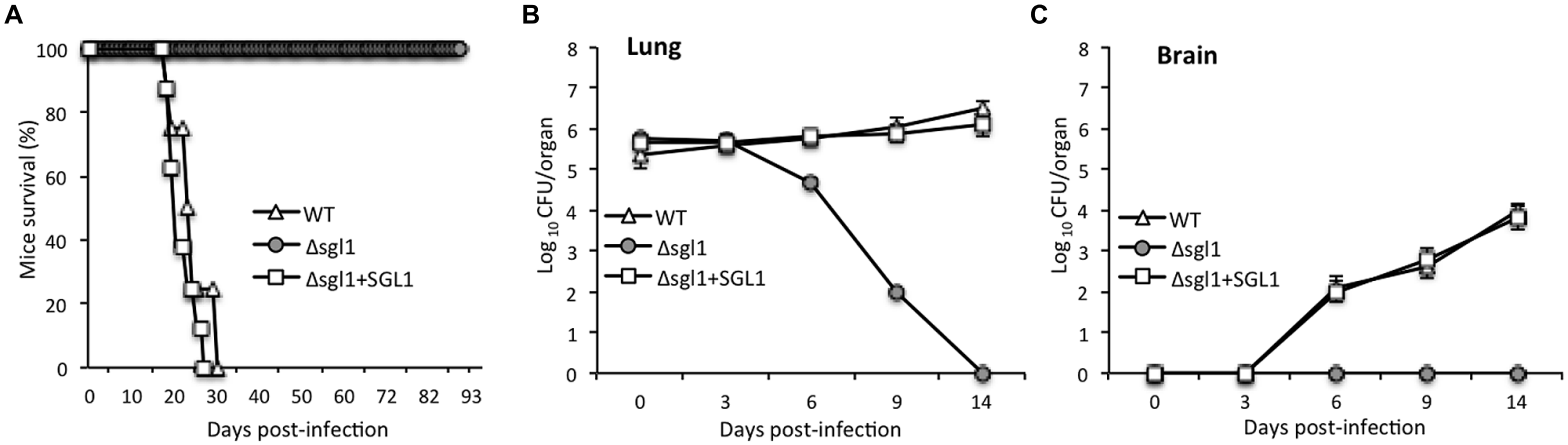
**Deletion of the *SGL1* gene in *C. neoformans* abolishes virulence. (A)** Virulence studies showed that 100% of mice infected with Δ*sgl1* survived the infection whereas mice infected with *C. neoformans* WT H99 or with Δ*sgl1+SGL1* reconstituted strain succumbed to infection within 24 ± 6 and 21 ± 7 days, respectively; *n* = 8 mice in each group. **(B)** Lung tissue burden analysis showed that the Δ*sgl1* mutant is eliminated from the lungs after 14 days of inoculation; *n* = 3 mice each at time point. **(C)** Brain tissue burden analysis showed that Δ*sgl1* is not found in the brain at anytime during the course of experiment; *n* = 3 mice at each time point.

Alterations in sphingolipid metabolism have been shown to attenuate cryptococcal virulence (Rittershaus et al., 2006; Singh et al., 2012). Thus, the virulence of the Δ*sgl1* strain in the mouse model of cryptococcosis was tested. Mice were infected with a lethal dose of fungal cells (5 × 10^5^ cells) to establish cryptococcosis and monitored for their survival. The average survival of mice infected with the WT *C. neoformans* was 24 ± 6 days whereas all mice infected with Δ*sgl1* strain remained alive during the course of the experiment (i.e., 90 days post-infection). Mice infected with the Δ*sgl1+SGL1* strain showed a survival pattern similar to that observed in the WT (average survival of 21 ± 7 days; **Figure 3A**). During the course of infection, lungs and brains were removed from the mice infected with the three strains and analysis of tissue burden was performed at days 0, 3, 6, 9, and 14 post-infection.

Interestingly, the number of Δ*sgl1* cells in the lungs decreased starting at day 3 and continued until day 14, at which point the lungs were completely clear of fungal cells (**Figure 3B**). Furthermore, no Δ*sgl1* cells were observed in the brain (**Figure 3C**), suggesting that fungal cells did not disseminate to the brain in the Δ*sgl1-*infected mice. In contrast, a significant number of fungal cells were found in the lungs and brains of mice infected with the WT or the Δ*sgl1+SGL1* strain (**Figures 3B,C**). In both cases, the number of fungal cells in the brain increased as a function of time, demonstrating the occurrence of extrapulmonary dissemination and progression of the disease. The findings of the tissue burden studies were confirmed by lung and brain histology observations, which showed no fungal cells in the organs isolated from the Δ*sgl1*-infected mice at the end of the experiment, but significant tissue damage and presence of fungal cells in the WT or Δ*sgl1+SGL1* strains (Supplementary Figure S4). These experiments reveal that sterylglucosidase is a virulence factor in *C. neoformans*, as the loss of this enzyme leads to loss of virulence in the mouse model.

### The Δ*sgl1* Strain Acts as a Vaccine Against Cryptococcosis in the Mouse Model

*Cryptococcus neoformans* cells possess a number of virulence factors that contribute to their survival inside the host, resistance to immune response, and detrimental activity against the host (Coelho et al., 2014). To gain more insight into the loss of virulence of the Δ*sgl1* strain, a number of virulence factors in this strain were evaluated and compared to the WT. In comparison to the WT, the Δ*sgl1* strain showed similar growth in acidic and neutral pH (at 37°C and in the presence of 5% CO_2_), similar melanin production and capsule thickness, and no major difference in growth under oxidative or nitrosative stress. In addition, the WT and mutant strains showed similar intracellular growth during *in vitro* infection of the J774.16 macrophage-like cells (Supplementary Figure S5). These analyses suggest that the most common virulence factors are similar between the Δ*sgl1* strain and the WT denoting a different mechanism for the loss of virulence.

**FIGURE 4:**
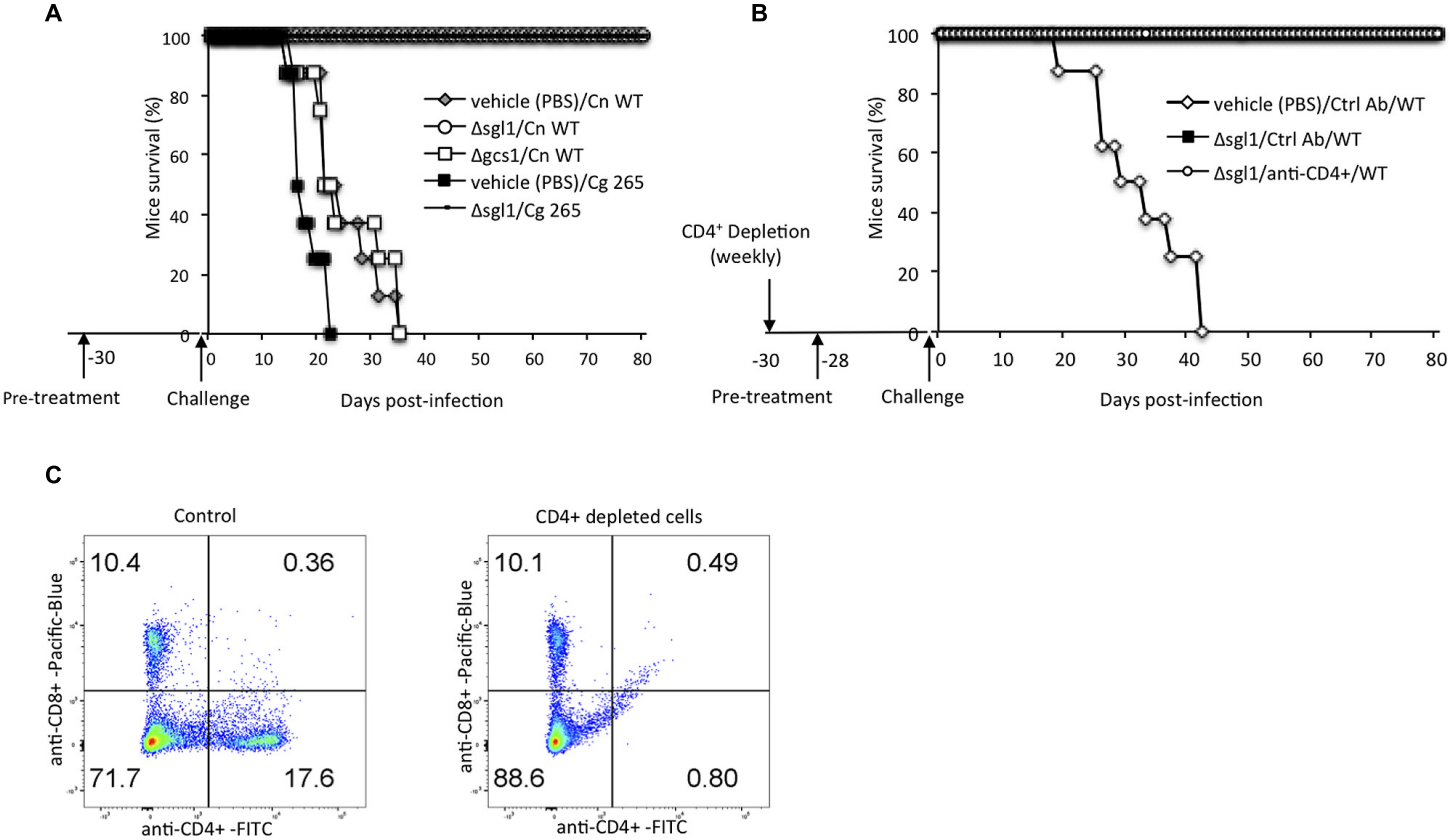
**Vaccination studies. (A)** Mice were “pre-treated” with Δ*sgl1* and, after 30 days (time 0), challenged with a lethal dose of *C. neoformans* wild-type H99 (*Cn* WT) or *Cryptococcus gattii* WT R265 (*Cg* 265). Mice exposed to Δ*sgl1* remained alive during the course of experiment (80 days post-infection) whereas all mice that were not exposed to Δ*sgl1* but to vehicle (PBS) or to the Δ*gcs1* mutant strain and then challenged with *Cn* WT succumbed to infection within 35 days; *n* = 8 mice in each group. **(B)** Pre-treatment with Δ*sgl1* completely protected CD4^+^ T-cell depleted mice from a subsequent lethal challenge with *C. neoformans* wild-type H99 (WT); *n* = 8 mice in each group. Depletion of CD4^+^ was achieved by administering anti-CD4 (Ab) weekly during the entire course of the experiment. The depletion was confirmed by flow cytometry performed at the day of *Cn* challenge **(C)**. Results are representative of three separate experiments showing similar results.

Given that the Δ*sgl1* strain was non-pathogenic and that SGs are known immunostimulators (Lee et al., 2007; Grille et al., 2010), the potential use of the Δ*sgl1* strain as a vaccine against cryptococcosis was investigated. Two controls were used for these studies: a vehicle (sterile PBS) and the *C. neoformans* Δ*gcs1* strain (Rittershaus et al., 2006), which is avirulent, but does not accumulate SGs. Mice were infected with the vehicle, or 5 × 10^5^ cells of the Δ*gcs1* or the Δs*gl1* strains and after 30 days were challenged with a lethal dose of the virulent WT *C. neoformans* or *C. gattii* R265 strains. Interestingly, the mice that were pre-treated with the Δ*sgl1* strain were completely protected against the subsequent infection. However, the mice that were pre-treated with the vehicle or the Δ*gcs1* strain succumbed to infection within 35 days (**Figure 4A**). These results suggest that the Δ*sgl1* strain may stimulate a host immune response that successfully kills Δ*sgl1* and makes the host resistant to subsequent cryptococcosis.

Although *Cryptococcus* infections can aﬄict immunocompetent individuals, the majority of the population at risk, are those suffering from immune suppression, such as HIV/AIDS patients. A reduction in CD4^+^ T-cells in this population results in aggressive cryptococcosis, which can be life threatening (Jarvis et al., 2013). The efficiency of the Δ*sgl1* strain as a vaccine against cryptococcosis during immune suppression was examined by its administration in CD4^+^ T-cells depleted mice prior to infection with the WT *C. neoformans*. For these studies, mice were depleted of CD4^+^ by weekly administration of anti-CD4^+^ antibody or control antibody (rat IgG2b) starting a month prior to infection with WT *C. neoformans* (**Figure 4B**). A 94.3% percent reduction in CD4^+^ T-cells was achieved as confirmed by flow cytometry (**Figure 4C**). The Δ*sgl1* strain or control (PBS) were also administered to mice a month prior to infection. Mice were then infected with 5 × 10^5^ cells of the virulent WT *C. neoformans*. All mice that received the PBS and the antibody control succumbed to infection in 41 days, while all the CD4^+^ T-cells depleted mice that were vaccinated with the Δ*sgl1* strain survived the infection, demonstrating that this strain is not infectious and can protect immune suppressed mice against a subsequent cryptococcal infection (**Figure 4B**).

## Discussion

In the current study, a mutant *C. neoformans* strain lacking the enzyme sterylglucosidase 1 (Sgl1) was engineered and characterized. This strain accumulated SGs and was not virulent in the mouse model, despite not demonstrating any deficiency in commonly known cryptococcal virulence factors. Importantly, this strain acted as a vaccine and was able to protect CD4^+^ T-cells depleted and immunocompetent mice against subsequent infections with lethal doses of *C. neoformans* or *C. gattii*.

Deletion of the Sgl1 enzyme was used as a strategy to accumulate SGs in *C. neoformans.* The presence of this enzyme was recently reported in *C. neoformans* and found to hydrolyze both SGs and short chain glucosylceramide (Watanabe et al., 2015). However, our results provided a more thorough characterization because it included physiologically relevant glucosylceramide species, which are produced by *C. neoformans* cells. We found that Sgl1 does not metabolize long chain glucosylceramide but only SGs.

The Δ*sgl1* mutant was not virulent in the mouse model of cryptococcosis. In the Δ*sgl1* strain, fungal cells were killed in the lungs within 14 days post-infection. No fungal cells were found in the brain tissue suggesting that the infection is completely controlled by the host in the lungs. It is important to note that the reduction in the fungal burden in the lungs starts at 3 days post-infection. This is due to the fact that the Δ*sgl1* strain does not show an appreciable growth defect compared to the WT and exhibits a similar resistance profile to stresses. Thus, the delay in the reduction of fungal burden is due to the time it takes for this strain to stimulate the immune response, which then kills the strain *in vivo*. Given that the loss of Sgl1 does not affect the growth profile or the common virulence factors of *C. neoformans*, this enzyme appears to be a stand-alone factor in controlling cryptococcal virulence. This is confirmed by the observation that the virulence phenotype was restored in the reconstituted strain (Δ*sgl1+SGL1*). The Sgl1 enzyme was active at physiological temperature and in the pH range of 4.5 to 8. The maximum activity was observed at pH = 4.5 after which the activity was sharply reduced with increased alkalinity. These activity profiles suggest that Sgl1 is highly active in the acidic environment inside alveolar macrophages; the activity is also present, although significantly reduced, in slightly acidic/neutral environment within the alveolar spaces.

Administration of the SGs accumulating Δ*sgl1* strain proved to be an effective vaccination strategy in the mouse models of cryptococcosis. When administered 30 days prior to the challenge, this strain was able to protect mice against subsequent infections with either WT *C. neoformans* H99 and *C. gattii* R265. These findings suggest that protection by the Δ*sgl1* strain is not serotype specific. Protection against the *C. gattii* R265 is of particular interest. The immune response against this strain is different from *C. neoformans* (Dong and Murphy, 1995; Wright et al., 2002; Cheng et al., 2009) and it has been the subject of a recent study for vaccination (Chaturvedi et al., 2014). It is important to note that the Sgl1 enzyme is capable of hydrolyzing cholesterol glucoside as well. Thus, although not experimentally tested, it is also possible that infection with this strain might lead to the uptake and accumulation of cholesterol glucoside from the host, which would be otherwise metabolized by the WT *C. neoformans*. However, it appears unlikely that cholesterol glucoside accumulation would contribute to immunity against the infection. The bacterium *Helicobacter pylori* routinely accumulates cholesterol glucosides during the infection of the mammalian host without eliciting any form of lipid-induced immunity (Wunder et al., 2006).

The Δ*sgl1* strain was also able to protect CD4^+^ T-cell depleted mice against cryptococcosis. SGs have been shown to enhance T-cell proliferation (Bouic et al., 1996) and promote Th1 immune response (Lee et al., 2007). A switch to Th1 cell-mediated immunity is the predominant mode of immune response against cryptococcosis (Decken et al., 1998; Koguchi and Kawakami, 2002; Wormley et al., 2007). In fact, promotion of Th1 response with an engineered interferon-gamma producing *C. neoformans* has been shown to protect mice against subsequent *C. neoformans* infections. Our results suggest that the immune stimulation by SGs goes beyond the Th1 immune response because our vaccination strategy is still effective in condition of CD4^+^ T cell deficiency. This is particularly advantageous as cryptococcosis mostly occurs in patients with this type of immune deficiency. It should be noted that while the Δ*sgl1* strain provides complete immunity in the mouse model of cryptococcosis, the administration of a live attenuated vaccine is associated with challenges such as undesired secondary mutations, which might revert the virulence (Teng et al., 2013). However, this strain can be used as a valuable starting point for the development of other vaccine formulations such heat-killed strains or vesicle formulations containing SGs, which would eliminate the dangers of using a live vaccine.

The immunomodulatory properties of SGs have been documented not only in the context of fungal infections (Lee et al., 2007), but also in other human diseases such as rhinitis, sinusitis, and tuberculosis (Bouic et al., 1996; Donald et al., 1997; Bouic, 2001). Although the underlying molecular mechanisms remain largely unknown; an increase in the level of T cells and cytokines induced by SG administration has been reported (Bouic et al., 1996; Donald et al., 1997). Although our data clearly show that the Δ*sgl1* strain accumulates SGs, we cannot rule out the presence of parallel/consequential mechanisms for the Δ*sgl1*-induced immunity. It is possible that other factors such as stimulation of CD4^+^ and CD8^+^ T cells are involved. The immunological basis of protection provided by vaccination with the Δ*sgl1* strain is an interesting topic for future studies.

## Conclusion

We have provided a comprehensive biochemical study of Sgl1, characterized the *C. neoformans*Δ*sgl1* strain and reported that the Sgl1 enzyme is a virulence factor in *C. neoformans*. The Δ*sgl1* strain is non-pathogenic and can protect immunocompromised mice against cryptococcosis. These results could have important implications for future vaccine development for cryptococcosis and other fungal infections.

## Author Contributions

AR and MDP conceived the study. AR designed and performed the majority of the experiments, analyzed the results and helped with writing the paper. VM helped in the design of the study and the experimental work on animals. AF contributed to data analysis and the design of experiments and wrote the paper. AS performed and analyzed the mass spectrometry experiments. AAS performed and analyzed the flow cytometry experiments. EI, NC, and MM provided critical help with the study design. CL helped with the experiments and provided a critical review of the study design. MDP contributed to the design of the experiments, analysis of data, and writing and reviewing the paper. All authors reviewed the results and approved the final version of the manuscript.

## Conflict of Interest Statement

The authors declare that the research was conducted in the absence of any commercial or financial relationships that could be construed as a potential conflict of interest.
