# Supplementary material for: Role of Sterylglucosidase 1 (Sgl1) on the pathogenicity of Cryptococcus neoformans: potential applications for vaccine development
Source: Front Microbiol. 2015 Aug 11;6:836. doi: 10.3389/fmicb.2015.00836 (PMC4531891; doi:10.3389/fmicb.2015.00836)
Supplement: Supplementary file 1 [file Presentation_1.PDF]

Supplementary Material
Role of Sterylglucosidase 1 (Sgl1) on the pathogenicity of *Cryptococcus neoformans*: potential applications for vaccine development

Antonella Rella[1], Visesato Mor[1], Amir M. Farnoud[1], Ashutosh Singh[1], Achraf A. Shamseddine[2], Elitza Ivanova[1], Nicholas Carpino[1], Maria Teresa Montagna[3], Chiara Luberto[4], and Maurizio Del Poeta[1,*]

[1]Department of Molecular Genetics and Microbiology, Stony Brook University, Stony Brook, NY 11794, [2]Department of Medicine, Stony Brook University, Stony Brook, NY 11794, [3]Department of Biomedical Science and Human Oncology, Hygiene Section, University of Bari, Italy, [4]Department of Physiology and Biophysics, Stony Brook University, Stony Brook, NY. 11794.

#Running Title: Sterylglucosides-accumulating *Cryptococcus* as fungal vaccine

***Correspondence**:
Dr. Maurizio Del Poeta,
Stony Brook University,
Department of Molecular Genetics and Microbiology,
145 Life Sciences Building, Stony Brook, NY, USA, 11794
Tel.: (631) 632-4024; Fax: (631) 632-9797;
E- mail: maurizio.delpoeta@stonybrook.edu

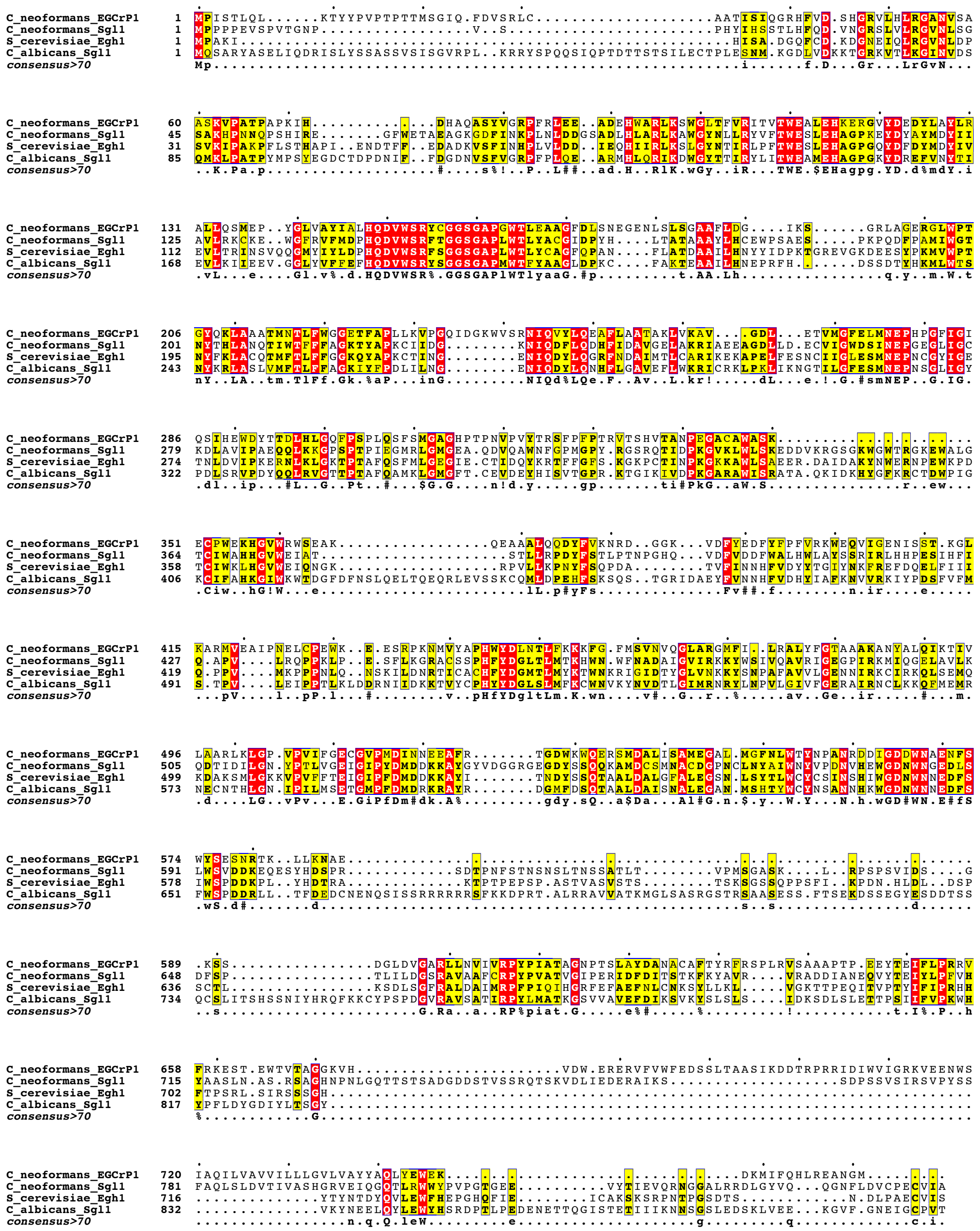

Supplementary Figure 1. Alignment of the entire protein sequences of *C. neoformans* protein EGCrP1, *C. neoformans* protein Sgl1, *S. cerevisiae* protein Egh1 and *C. albicans* protein Sgl1 (not characterized yet). Identical conserved residues are shown in red, homologous residues in at least 3 of the sequences are shown in yellow. The protein homology scores were 45% for *C. neoformans* EGCrP1 and *C. neoformans* Sgl1, 42% for *C. neoformans* EGCrP1 and *S. cerevisiae* Egh1, and 45% for *C. neoformans* EGCrP1 and *C. albicans* putative Sgl1 .

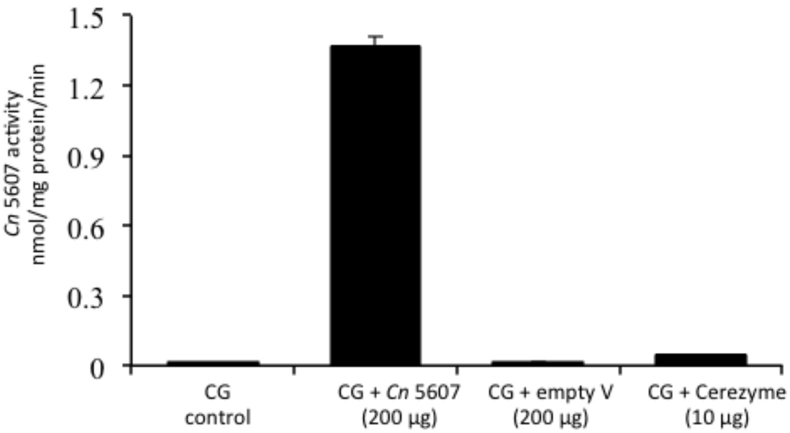

Supplementary Figure 2. CNAG_05607 enzyme has cholesterol glucosidase activity. The release of cholesterol from cholesterol glycoside by CNAG_05607 enzyme was monitored using GC-MS. Empty vector and Cerezyme were used as negative control. CG, Cholesterol glucoside; Empty V, empty vector.

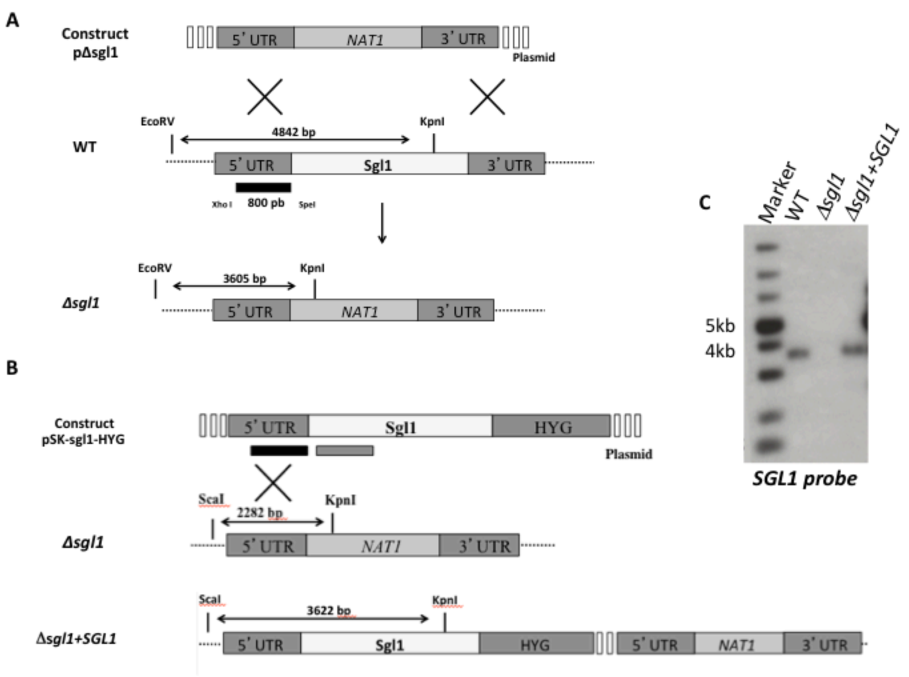

Supplementary Figure 3. Deletion and reconstitution of the *SGL1* gene. (A) Strategy for the deletion of *SGL1* in *C. neoformans* wild-type (WT) and creation of the mutant strain Δ*sgl1*. (B) Strategy for the generation of the complemented strain Δ*sgl1+SGL1*. (C) Southern Blot hybridization analysis of genomic DNA of WT, Δ*sgl1* and Δ*sgl1+SGL1* digested with ScaI and KpnI and screened with the "SGL1" probe (gray bar in B). Results show that the *SGL1* gene has been deleted in the Δ*sgl1* mutant and reconstituted in the Δ*sgl1+SGL1* strain. Gene deletion was confirmed using a second probe (5' UTR – black bar in B). 5' UTR, 5' untranslated region; 3' UTR, 3' untranslated region; NAT1, nourseothricin 1; Sgl1, sterylglucosidase 1; HYG. Hygromycin B.

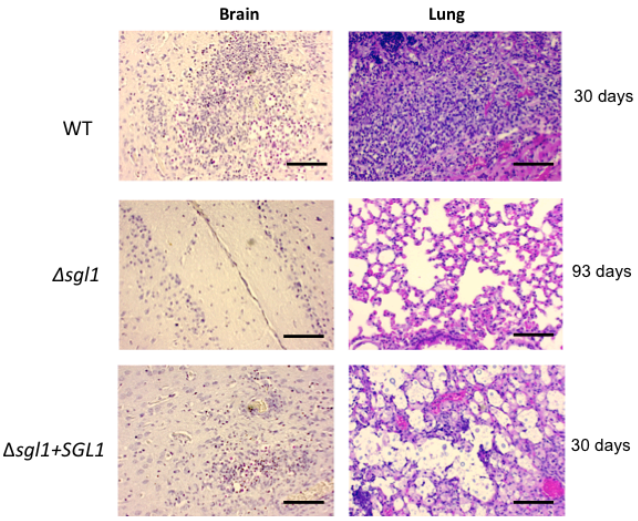

Supplementary Figure 4. Histopathology of brains and lungs obtained from the CBA/J mice infected intranasally with *C. neoformans* wild-type (WT), Δ*sgl1,* and Δ*sgl1+SGL1* strains. The brains were stained with mucicarmine stain, the lungs were stained with Hematoxylin and Eosin (H&E) stain. Black bar, 50 µm.

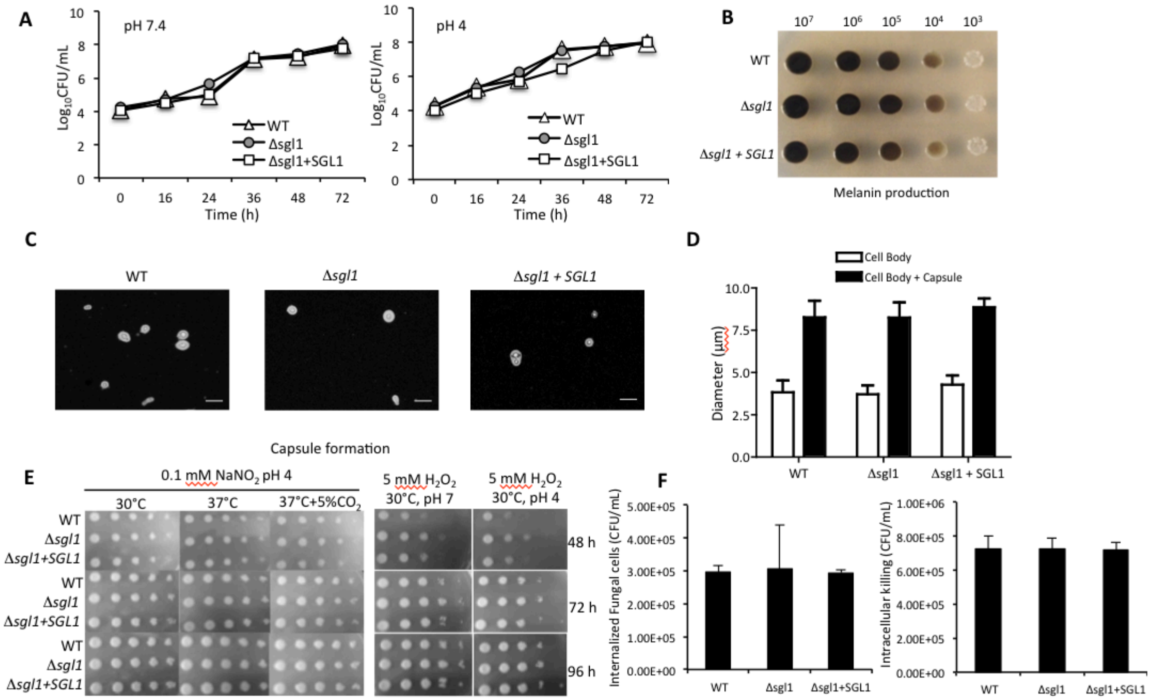

Supplementary Figure 5. Characterization of virulence phenotypes in the Δ*sgl1* mutant strain. A) *C. neoformans* wild-type (WT), Δ*sgl1,* and Δ*sgl1+SGL1* strains showed identical growth in DMEM at 37°C, 5%CO_2 and at pH 7.4 or pH 4. B) *C. neoformans* cells were plated on medium containing L-DOPA and the melanin produced by WT, Δ*sgl1,* or Δ*sgl1+SGL1* strain was similar. C) Capule size of WT, Δ*sgl1,* or Δ*sgl1+SGL1* strains was similar when cells were incubated in DMEM at 37°C, 5%CO_2. White bar, 10 μm D) Cell body and capsule size of WT, Δ*sgl1,* or Δ*sgl1+SGL1* strains, reported by measuring the cell body and capsule size of 50 cells for each strain under the microscope. E) There were no major differences among WT, Δ*sgl1,* and Δ*sgl1+SGL1* in their ability to grow under stressed conditions (hydrogen peroxide and nitrosative stress). F) Phagocytosis and intracellular killing within a macrophage-like cell line J774A.16 was identical between WT, Δ*sgl1*, or Δ*sgl1+SGL1* strains at 2 and 24 hours, respectively.
